# In situ feeding as a new management tool to conserve orphaned Eurasian lynx (lynx lynx)

**DOI:** 10.1002/ece3.7261

**Published:** 2021-03-02

**Authors:** Joe Premier, Martin Gahbauer, Franz Leibl, Marco Heurich

**Affiliations:** ^1^ Albert Ludwig University Freiburg Germany; ^2^ Department of National Park Monitoring Bavarian Forest National Park Grafenau Germany; ^3^ Leibniz Institute for Wildlife and Zoo Research Berlin Germany

**Keywords:** carrion, conservation management, large carnivore conservation, orphan juvenile, species reintroduction, supplementary feeding

## Abstract

High human‐caused mortality due to wildlife‐vehicle‐collisions and illegal killing leads to frequent cases of orphaned Eurasian lynx juveniles. Under natural conditions, this would result in starvation of the young. To avoid this, wildlife managers conventionally rear animals in captivity and release them later. However, this measure is an undesirable outcome for species conservation, managers, and animals alike. Increased awareness of Eurasian lynx orphaned by human‐caused mortality means managers must often intervene in endangered populations. In this study, we report for the first time a successful case of in situ feeding designed to avoid captivity of two orphaned Eurasian lynx. We exposed 13 roe deer and 7 red deer carcasses in the field to successfully support two orphans to the age of independence and confirm dispersal from the natal range. We present this management approach as a feasible and complimentary tool that can be considered in small or isolated large carnivore populations where every individual counts toward population viability.

## Graphical abstract

The topic of orphaned juvenile Eurasian lynx is one of growing importance. Given the high rates of human‐caused mortality of Eurasian lynx, especially in Central Europe's anthropogenic landscapes, large carnivore managers must frequently deal with orphans. It is necessary to intervene in order to conserve individuals but the options available to managers besides conventional captive rearing have been relatively unexplored. We describe how two juvenile Eurasian lynx became orphaned and could be supported for 5 months to independence age via in situ provisioning of deer carcasses, thus avoiding human contact and captivity. We also confirmed dispersal from the natal range and the success of this method.

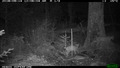

## 1 INTRODUCTION

In recent years, the occurrence of orphaned Eurasian lynx *Lynx lynx* (hereafter lynx) has been increasingly attracting the attention of large carnivore and conservation managers. Under pressure from growing traffic volumes, flushed out of their den refuges during wood extraction and due to human persecution, mothers are frequently taken from their litters. In the last decade at least 80 orphaned or displaced lynx have been reported, with a peak of 14 orphans occurring in 2016 (Jobin‐Molinari, Personal communication).

Most lynx populations in Central Europe are the result of a political shift induced by the shifting attitudes of citizens (Chapron et al., 2014). In the wake of this, a wave of reintroduction projects followed in the 1970s (e.g., Cop & Frkovic, 1998; Vandel et al., 2006; Wölfl et al., 2001). Since founding these populations have unfortunately remained relatively small and isolated, which increases their risk to demographic stochasticity and Allee effects (Bull et al., 2016). Additionally, the limited number of founders and lack of genetic exchange increases the likelihood of inbreeding and potential fixing of deleterious genes via drift (Sindičić et al., 2013). A priori, juveniles are vital to avoid extinction and genetic bottlenecks. Their importance for genetic diversity goes beyond this, as any potential population exchange might rely on natal dispersal, for which enough juveniles are prerequisite. Therefore, the importance of each juvenile in these reintroduced populations cannot be overstated.

Typically, orphaned lynx come to the attention of managers following interactions with humans, vehicles, or livestock (Breitenmoser‐Würsten et al., 2007; Schmidt‐Posthaus et al., 2002). This could be driven by hunger‐mediated risk‐taking (e.g., Blecha et al., 2018) or simply maladaptive naivety toward threats. Conventionally, this results in the capture of these individuals. Since lynx in Central Europe are born around May and independence is normally observed 10 months later around February (Breitenmoser‐Würsten et al., 2001), capturing a juvenile lynx means keeping them in captivity until spring (Wilson et al., 2019). Keeping wild animals in captivity is not trivial and has potentially deleterious outcomes (Lane & McDonald, 2010). These might include physical, physiological, or psychological trauma, which might not be apparent until release. Furthermore, once a captive lynx reaches maturity a suitable release site must be found. Finding a release site where there is agreement from all stakeholders present is also not trivial. In populations where trapping projects are ongoing (i.e., source populations for reintroduction or reinforcement projects), such orphans are a useful source of animals (Kubala et al., 2019). However, in reintroduced populations where each offspring's survival is critical for population viability a sensitive and careful approach should be taken, considering the needs of the orphans, stakeholders, and the wider conservation context of the target species.

Here, we present a case study where, to the best of our knowledge for the first time, in situ feeding was used to support the rearing of two free‐ranging orphaned lynx whose mother was killed in a vehicle collision in the Bavarian Forest National Park.

## 2 MATERIALS AND METHODS

**FIGURE 1:**
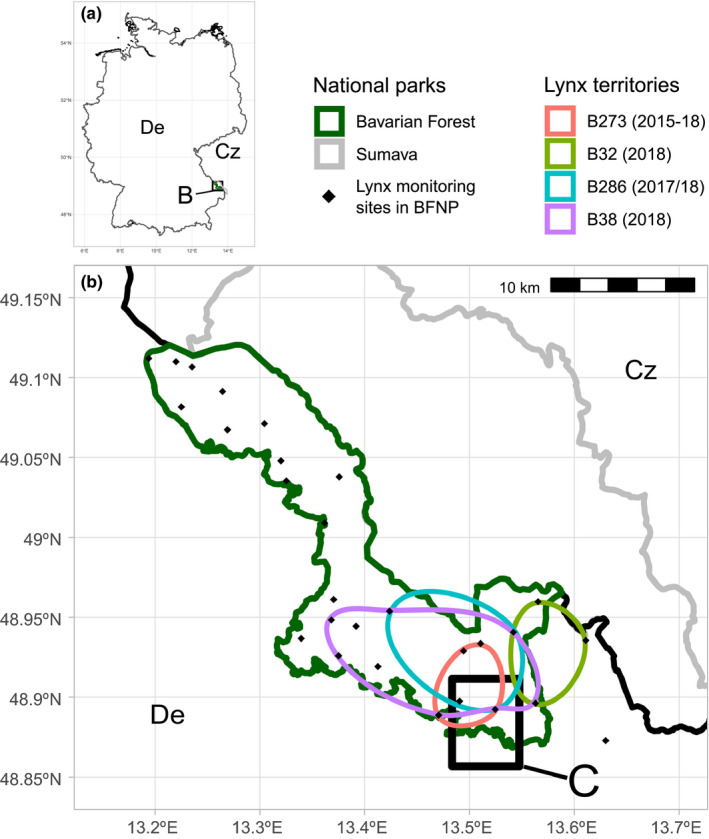
Location of the study area (C – Figure 2) in the Bavarian Forest national park, Germany (De) and the adjoining Sumava national park, Czechia (Cz). Including approximate territories of relevant lynx determined via camera trap monitoring (BFNP sites only): B273 (mother of B286 last observed 27/01/2018), B286 (mother of orphans), plus resident males B38, and B32

The in situ feeding of two orphaned juveniles was conducted opportunistically following the death of their mother “B286.” B286 was a resident adult born in 2016 and part of the Bohemian‐Bavarian‐Austrian lynx population (BBA). The BBA follows the Czech‐German border from the Erzgebirge/Krušné hory in the north, down to the Waldviertel, Austria in the south. Following its reintroduction in the 1980s, the BBA expanded to fill its current range increasing from 17 released individuals to approximately 80 in 2014 (Cerveny et al., 1996; Wölfl et al., 2001). The population has somewhat stagnated in recent years, in part due to high rates of vehicle collision, but also likely due to poaching (Heurich et al., 2018). B286’s territory was situated partially within the Bavarian Forest National Park, Germany, and partially outside in the cultural landscape (Figure 1). This territory she inherited from her own mother, B273, who was last observed on 27/01/2018 (Bavarian Forest and Sumava National Parks, 2018). The human density in this region is low at approx. 2 and 70 inhabitants/km^2^ inside and outside the national park, respectively (Germany average = 227 inhabitants/km^2^); however, there are unfenced major roads in the park's vicinity.

**FIGURE 2:**
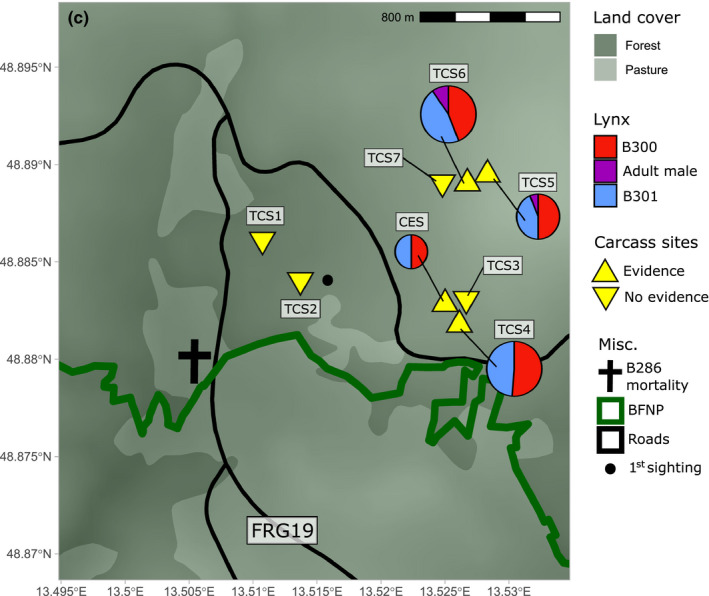
Spatial schematic of carcass exposures in the region of orphaned lynx activity and the collision site. Carcasses exposed at CES were part of a carrion ecology project, while sites TCS3,4,5,6, and 7 were targeted carcasses (TC). Two carcasses which were not consumed (at TCS1 and 2) were relocated to TCS3. Pie charts indicate proportion of days with visits for each individual, with the radii indicating total number of visits by all individuals (see Figure 3)

In the national park, a systematic camera trap monitoring program is underway (Weingarth et al., 2012). In this way, following reports of a lynx vehicle collision, the identity of the victim B286 could be determined in the field. The collision took place at approx. 19:00 on 14/10/2018 on the FRG19, a county‐level road, near “Lichtau” (Figure 2). In the weeks prior to the accident, local hunters had reported sightings of a mother with kittens at the same location (sighting by local hunter 13.10.2018 at 22:00 reporting 3 kittens and mother) and camera trap images had confirmed presence of at least 2 kittens, but the mothers’ identities could not be confirmed. Furthermore, although B286 was not photographed with any kittens her physical appearance in camera trap photographs suggested motherhood. Following the accident, these factors led to the immediate assumption that orphaned juveniles must be in the area. Given the death in mid‐October and the average birth date of lynx in May, it could be assumed that the juveniles were 5 months old and had not yet reached independence. Therefore, we opportunistically began an in situ feeding program.

We exposed 15 shot or road‐killed deer carcasses close to evidence of the orphans (camera trap photographs, sightings) between 15/10/2018 and 13/02/2019, so‐called “targeted” carcasses (TC). The last “targeted” carcass was exposed 121 days after B286’s death. In addition to this opportunistic feeding, throughout the period an existing carrion ecology research project in the national park was underway (Ray et al., 2014; Stiegler et al., 2020). In the scope of this project, 5 deer carcasses, abbreviated as CE, were coincidentally exposed at a single long‐term site (CES). Over the period, 8 different sites in the vicinity were used to expose a total of 20 carcasses, 13 roe deer (*Capreolus capreolus*), and 7 red deer (*Cervus elaphus*), for the orphans to feed upon (Table 1).

**Table 1:** Roe deer (r) and red deer (R) carcasses exposed in the vicinity of orphaned lynx, as part of a carrion ecology project (CE) and targeted in situ feeding (TC), at site CES and TCS1‐7 respectively

Date exposed	TCS 1	TCS 2	CES	TCS3	TCS4	TCS5	TCS6	TCS7	*Totals*
15/10/2018	r_1_	r_2_	r						*3r*
23/10/2018				r_1_ + r_2_					*2r*
25/10/2018			r			r			*2r*
01/11/2018						r			*r*
05/11/2018						r			*r*
06/11/2018			R						*R*
13/11/2018						r			*r*
17/11/2018				R					*R*
21/11/2018					R	r			*R + r*
10/12/2018						r			*r*
18/12/2018							R		*R*
07/01/2019							r		*r*
15/01/2019			r				R		*r + R*
06/02/2019							r		*r*
13/02/2019								R	*R*
25/02/2019			R						*R*
***Totals***	*1r*	*1r*	*3r + 2R*	2r* + 1R	*1R*	*6r*	*2r + 2R*	*1R*	*13r + 7R*

Note

A total of 13 roe deer and 7 red deer carcasses were available over this period. Carcasses r_1_ and r_2_ exposed on 15/10/2018 at sites TCS1 and TCS2 were relocated on 23/10/2018 to site TCS3, indicated with * in site total.

Initially, two roe deer TCs were exposed in a forest patch close to the location of B286’s death and a roe deer CE was exposed at the CES, approx. 500m east (Figure 2). These were intended to encourage the orphans to move away from the area of high traffic density and deeper into the national park. Following this, a further 19 deer carcasses over a period of 4 months were exposed among various sites in an approx. 0.37 km^2^ area within the national park boundaries. The sites (Figure 2) and cadaver exposure timing were made to promote exploration behavior in the orphans, especially into the heart of the relatively safe national park. We monitored TC1, TC2, and all CEs using automatic wildlife cameras (Reconynx HC600 Hyperfire) and visited these carcasses daily (TC1,2) and monthly (CEs) to check for evidence of feeding. All other TC sites (TCS) were monitored using GSM enabled automatic wildlife cameras (Dörr Snapshot Mobil 5.1). Images captured by these cameras were received by email, allowing continuous monitoring of the carcasses. In addition, the cameras allowed us to assess the presence of scavengers and, thanks to distinctive markings found on the pelts of lynx in this population, individually identify photographs of lynx (e.g., Weingarth et al., 2012).

## 3 RESULTS

**FIGURE 3:**
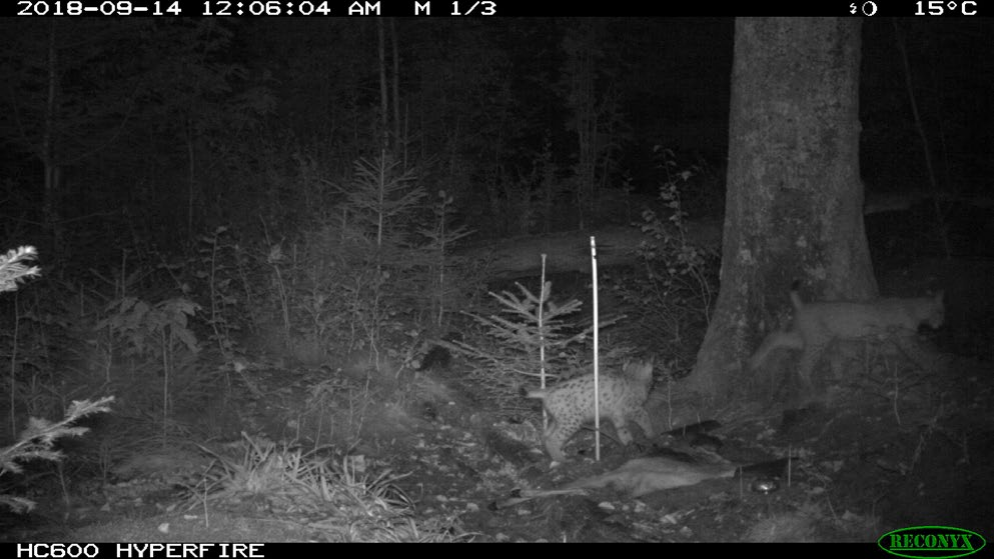
Photographic evidence of the orphans, large‐spotted B300 (left) and marbled B301 (right) at a carcass exposed at CES, in September 2018 before their mother's death

**FIGURE 4:**
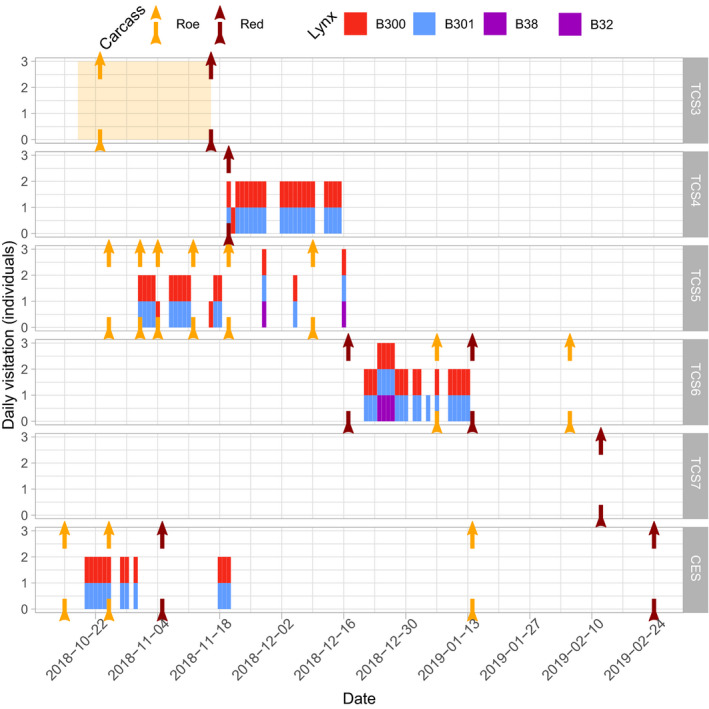
Schematic of roe and red deer carcass exposure timing (arrows) among 6 different sites (see Figure 3) and lynx visitation between 15/10/2018 and 25/02/2019. Carcasses exposed at CES were part of a carrion ecology project, while sites TCS3,4,5,6, and 7 were targeted carcasses. The images from the camera at TCS3 were lost but orphan presence was confirmed (yellow region). Adult male sightings before 01/12/2018 were B38, subsequently, they were B32

Following the mother's death (14 October), the first evidence of the two orphans was obtained on 18 October by a wildlife camera of another research project. The individuals could not be sexed but were individually identifiable as B300 (large‐spotted) and B301 (marbled) (Figure 3). The spatial and temporal distribution of lynx evidence and carcass exposures, described in the following, are found in Figures 2 and 4, respectively, and Table 1. The first two TC, two roe deer, were exposed the day after the mother's death in the vicinity of the accident. Since the first two TCs were left intact, that is, not visited by orphans nor consumed by other scavengers, they were removed on 23 October. A third roe deer carcass was placed at the CES on 15 October and received its first visit from the orphans on 20 October. This CE was visited by the orphans daily until another carcass was added to CES on 25 October, which was subsequently visited on three separate days. In the meantime, the two roe deer TC which were removed were relocated close to the CES (approx. 75m distance) at targeted carcass site (TCS) 3. A storage problem meant data from TCS3 were corrupted; hence, no temporally explicit information is available at this site, but orphan presence was confirmed visually before data were lost. Starting with a roe deer on 25 October at TCS5, carcasses were exposed in a cluster 800m northeast of the CES. Presence of the orphans at this cluster was confirmed on 1 November. A series of 5 roe deer were exposed at TCS5, despite which orphan occurrence began to reduce following the exposure of a red deer at TCS4. Thereafter, TCS4 was visited almost daily until 16 December. Two days later, on 18 December, a red deer was exposed at TCS6. This site was visited frequently for approximately 1 month, with just 1 roe deer added in that time. From 14 January until 25 February, a further 3 red deer (1 CE, 2 TC) and 2 roe deer (1 CE, 1 TC) were exposed among CES, TCS6, and TCS7. However, no further evidence of the orphans was collected by the camera traps. Since we define the natal range as the area of orphan activity and in situ feeding, apparent dispersal occurred sometime around mid‐January. This constitutes 10 months after the conventional lynx birthdate and almost half of this time (approx. 5 months) as orphans.

At TCS5 and TCS6 deer, carcasses were also visited by two adult male lynx. The adult males “B38” and “B32” were first observed in the national park in 2015 and 2014, respectively, and are known to be residents with overlapping territories, confirmed via camera trapping (Figure 1). B38 visited a carcass 1 day in November, while B32 was present 5 separate days in December. On all 6 days that an adult male visited the carcass sites, the orphans also visited.

**FIGURE A1:**
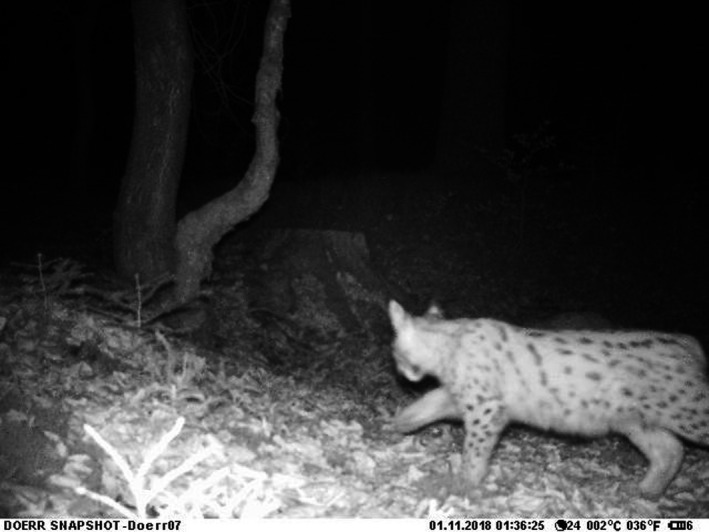
Evidence of B300 (large‐spotted) in November 2018

**FIGURE A2:**
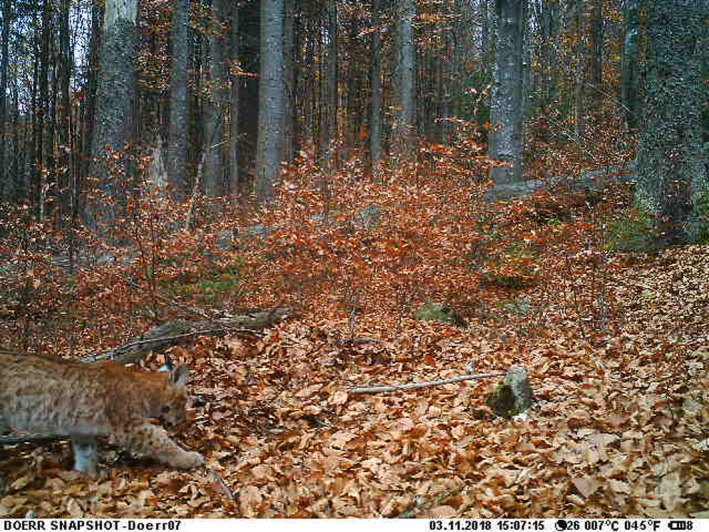
Evidence of B301 (marbled) in November 2018

**FIGURE A3:**
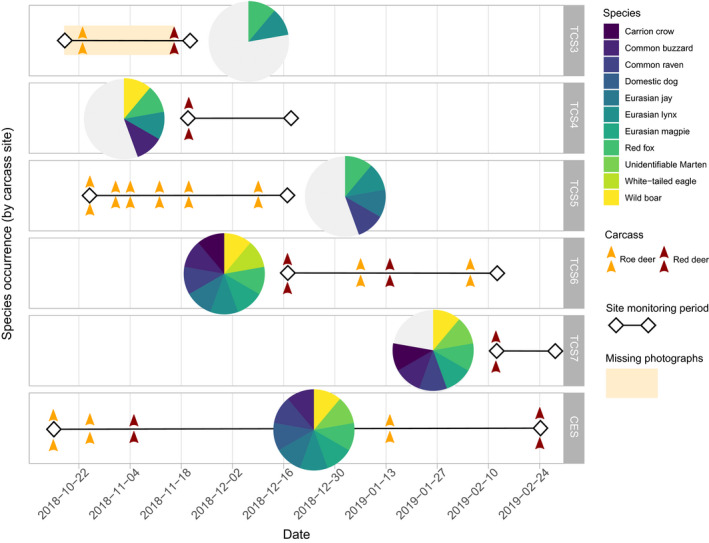
Schematic of carcass exposure timing, site monitoring periods, and scavenger species occurrence at the carcass sites

Consumption was directly observed via camera trapping and/or assumed following inspection of the carcasses. The orphans typically remained at a carcass for several hours per visit and tended to feed in series. Taking evidence from the CES (114 photographs), the site with the most appropriate camera, the activity of the orphans was crepuscular with 76% of photographs around dusk/dawn and 24% at night. On all but 3 days camera traps could capture both individuals at the same site (Figure 4). The good physical condition of the orphans was observed qualitatively in the photographs (Figures A1 and A2). Therefore, we can say with some certainty that the lynx orphans consumed and gained mass from the in situ feeding. Apart from lynx, the scavenger community was dominated by red fox (*Vulpes vulpes*), wild boar (*Sus scrofa*), and common raven (*Corvus corax*). Over the respective monitoring periods, a maximum of 9 scavenger species were observed at one site, with a total of 11 species among all sites (Figure A3). Less frequent scavengers, such as common buzzard (*Buteo buteo*) or white‐tailed eagle (*Haliaeetus albicilla*), were only observed at sites where red deer carcasses were exposed. The specific consumption rates of scavengers were not measured, and carcass attenuation was only monitored insofar as to maintain carrion availability.

**FIGURE A4:**
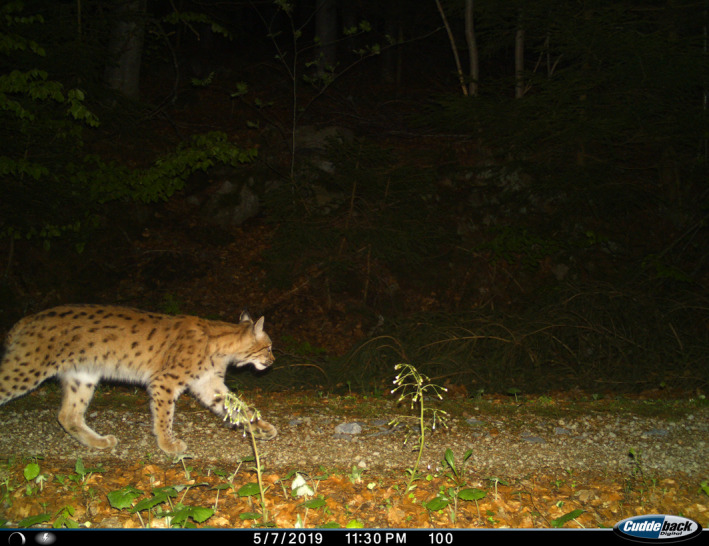
Evidence of B300 (large‐spotted) at independence in May 2019 close to Bodenmais, Germany, approx. 50 km from natal range. Image supplied by the Bavarian Environmental Agency (Bayerisches Landesamt für Umwelt)

**FIGURE A5:**
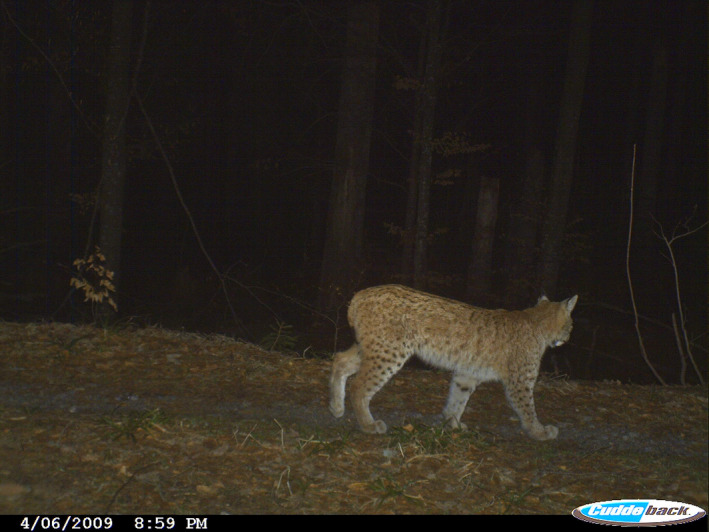
Evidence of B301 (marbled) at independence in April 2019 approx. 10 km from natal range (incorrect year on image due to problem with older generation Cuddeback cameras)

Evidence of B300 was collected by the Bavarian Environmental Agency (Bayerisches Landesamt für Umwelt) on 07/05/2019 in the region west of the national park (Figure A4). On 06/04/2019, photographic evidence of B301 was collected by a camera trap 10 km northwest of the orphans’ original area of activity (Figure A5). These can be taken as proof of survival to independence for both orphans. Monitoring is still ongoing in the region, though we have not received further evidence of the orphaned lynx since this time.

## 4 DISCUSSION

Lynx in the reintroduced populations of Central Europe suffer high mortality due to traffic and illegal killing (Sindičić et al., 2016, Heurich et al., 2018), which can frequently lead to orphaned juveniles. Conventionally, these orphans are captured and rehabilitated in captivity. In this study, we present a recent case of an opportunistic in situ feeding program designed to rear orphaned lynx juveniles to independence without direct physical intervention. After exposing 20 deer carcasses over 121 days in a 0.37 km^2^ area of their activity, dispersal from the natal range was apparent. We subsequently obtained evidence of the two orphans which was used to confirm survival to independence and success of the management action.

In Europe, it is uncommon to leave carcasses exposed in the wild; however, the practice is increasing due to supposed benefits for charismatic species and biodiversity (Fielding et al., 2014). In this study, we showed that carcass provisioning could also be a complimentary tool to conserve orphaned lynx. We were able to observe visitation of approx. 75% of exposed carcasses, and nearly all carcasses exposed before mid‐January. At half of the visited sites, the first exposed carcass took some days before the orphans discovered it and two sites were not visited at all. This leads us to believe the orphans were exploring the area, possibly motivated by the distribution of carcasses. The timing of apparent dispersal was relatively early compared to some literature, for example, in a study of the Northwest Alpine population juveniles first left their mothers in February and left her territory as late as September or October (Breitenmoser‐Würsten et al., 2001). The orphan's early departure means the orphans were sufficiently well‐fed and suggests they physically matured at a faster rate than normal. Independence and necessity for finding food probably encouraged a faster mental development and might also have contributed to early dispersal. Although this is not particularly desirable, or undesirable, it signals that the provisioning was enough (around 1 deer per week). A lower provisioning rate might result in more natural dispersal timing, but risking malnutrition of orphans should be avoided. The same could be said about spatial distribution, although exploration should be encouraged, the risk of orphans not finding food is best averted. These factors further depend on scavenging competitors, who might rapidly attenuate carcasses (e.g., Ray et al., 2014). Therefore, we recommend close monitoring of carrion availability.

Holding wild animals in captivity can negatively affect well‐being in diverse ways (Lane & McDonald, 2010). There is evidence that for wide‐ranging species, including large carnivores, that captivity has negative psychological effects and furthermore these can lead to deteriorating fitness (Clubb & Mason, 2003). Animals which have been reared in captivity are often believed to be habituated to humans, an undesirable outcome when these animals should be released into the wild later (Vandel et al., 2006). For many species, including lynx, juveniles in captivity might miss out on key developmental stages for which the mother is normally present and being fed in an enclosure might be detrimental to the hunting instinct (Sikes & Gannon, 2016). Reintroductions which are founded using animals raised in captivity have experienced variable successes. In one meta‐analysis by Jule et al. (2008), it was shown that captivity negatively affected the individual survival rate of carnivores in reintroduction projects.

Despite the literature which speaks to the dangers of captivity, for lynx there are many cases where captive‐reared animals have been successfully released into the wild. For example, the Harz Mountains population was entirely founded using captive‐bred lynx, although they were maternally reared (Anders & Sacher, 2005). This population has fared well in the last two decades and is considered stable (Mueller et al., 2020). Dispersing individuals have almost reached neighboring populations, which has even fuelled speculation of the greater propensity for exploration of captive animals. Furthermore, a current population reintroduction in the Vosges‐Palinatian region is making use of captive‐reared orphans (*n* = 6) translocated from the autochthonous Carpathian population. At this early stage, it appears the orphaned individuals are faring well having successfully hunted and reproduced (Kubala et al., 2019).

Practically speaking capturing orphans and keeping them in captivity presents many difficulties for managers (Miller et al., 1999). Enclosures for large carnivores must meet relevant ethical and legal criteria, such as size, materials, and enrichments, and they must be nearby and constantly available in case a management intervention requires it. Following construction costs, the resources required to run such an enclosure must always be in place. This must include trained and experienced rehabilitation personnel. These factors could make these facilities costly, although the same might be said for the in situ method presented. If the rate of orphans increases, new enclosures will be needed. Furthermore, from an ethical standpoint, managers have responsibility for these individuals from captivity until they are reasonably capable of surviving (Waples & Stagoll, 1997). A detailed review of captive lynx husbandry is one recommendation adopted by the European Council (Standing Committee of the Bern Convention, 2019).

The potential benefits of leaving orphans in the wild as in our case study methodology are several. First, ethical considerations regarding captive holding of wild animals can be minimized. Second, in terms of epidemiology, there is no risk of transferring diseases from captive to wild populations and vice versa. Third, rearing juveniles in the wild is closer to a natural state than captive rearing, for example: (a) no risk of inadvertent habituation, (b) feeding with carrion simulates natural scavenging which is a normal dietary component (e.g., Krofel et al., 2011), (c) juveniles have ample opportunity to practice hunting, and (d) better body condition thanks to more diverse habitat and space‐use. Finally, there is no risk of life‐time captivity of the animal, which might be the case if rehabilitation in an enclosure fails.

The successful application of our methodology in the future could depend on specific conditions. Our method relied heavily on human resources available in the study area, that is, hunters and field workers. We were able to supply carcasses of shot and road‐killed deer thanks to an ongoing carrion ecology project. This project necessitates the provision and cold storage of carcasses which cannot be assumed for ordinary wildlife management entities. However, in regions of lynx presence one might safely assume a priori an adequate supply of wild deer which might be hunted ad hoc for this purpose. A further factor which might limit broader appeal is the public perception of carrion (Dupont et al., 2012). The Bavarian Forest National Park, where this case study took place, has the freedom to leave carcasses to natural succession. Outside of protected areas, we would expect some resistance if management entities attempted to expose carcasses, which will depend on site or country‐specific social and legal restrictions. Given the number of species which depend on carrion, carcass provisioning in Europe is a subject which demands more attention and for orphans which are not detected the availability of natural carrion would be a huge boost to their survival chances. Finally, the circumstances of each case cannot be overlooked. Here with good infrastructure, we could support two 5‐month‐old (approx.) orphaned siblings, which implicates the importance of the mother's prior contribution and the siblings’ mutuality in their social and physical maturation as conditions to our intervention.

Management actions must be considered within a broader species conservation context. For example, source for reintroductions or reinforcements might find translocation of orphans as an attractive alternative to translocation of mature individuals (Kubala et al., 2019). The genetic impoverishment of Central European lynx populations means genetic management is beginning (e.g., Wilson et al., 2019). To this end, translocation of orphans among a network of reintroduced populations as part of meta‐population management could be considered, although the potential genetic benefits of such actions should be scrutinized in advance. Until then, we recommend our in situ rearing as a complimentary method primarily for application in isolated reintroduced populations where every individual counts for population viability and against inbreeding risk (Bull et al., 2016).

## 5 CONCLUSION

High rates of human‐caused mortality among large carnivores (Heurich et al., 2018; Sindičić et al., 2016) means managers are frequently called to deal with orphaned juveniles. The conventional tactic of captive rearing before re‐release has significant material and immaterial costs for managers and animals alike. Therefore, alternative methods should be explored in the future to avoid upsetting the well‐being of animals or risking the viability of at‐risk populations. The Eurasian lynx has been the subject of many management actions in the last five decades, yet the reintroduced populations still find themselves restricted in size and isolated. We offer evidence of in situ supplementary feeding of orphaned lynx as a feasible option that promotes a close to natural rearing of large carnivore juveniles. Future cases which employ this method, or other methods, should ensure rigorous monitoring post hoc so that the survival rates of orphans with different life histories might be ascertained and compared.

## CONFLICT OF INTEREST

None declared.

## AUTHOR CONTRIBUTION

**Joseph Premier:** Conceptualization (supporting); Formal analysis (lead); Visualization (lead); Writing‐original draft (lead); Writing‐review & editing (lead). **Martin Gahbauer:** Conceptualization (supporting); Investigation (lead); Writing‐review & editing (equal). **Franz Leibl:** Supervision (supporting); Writing‐review & editing (supporting). **Marco Heurich:** Conceptualization (lead); Supervision (lead); Writing‐original draft (supporting); Writing‐review & editing (equal).

## Data Availability

Carcass locations and camera trap observations: https://doi.org/10.5061/dryad.z08kprrbq
